# Book Reviews

**Published:** 1897-08

**Authors:** 


### BOOK REVIEWS.

Fracture of the Radius. By John B. Roberts, A.M., M.D., Professor of Anatomy and Surgery in the Philadelphia Polyclinic; Professor of Surgery in the Woman's Medical College of Pennsylvania, etc. Published by P. Blakiston, Son & Co., Philadelphia. Price $1.00.

This is "a Clinical, Pathological and Experimental Study of Fracture of Lower End of the Radius with Displacement of the Carpal Fragment *toward the Flexor or Anterior Surface* of the Wrist." The author first presents a number of illustrative cases and specimens; this is followed by some experimental observations on the cadaver, and then the causes and mechanism of this fracture are discussed. The treatment of these fractures and the methods of reduction and retention by splints (adhesive plaster, gypsum, plaster of Paris, wood, etc.) are fully described. There are thirty-three illustrations, including two X-ray pictures.

Fracture of the wrist is one of the commonest which the practitioner encounters, and while *backward* displacement of the carpal fragment is the usual deformity, "*forward* displacement also happens in a considerable number of instances." This monograph deals thoroughly with the subject from this point of view, and the practitioner will find it exceedingly instructive.

---

Manual of Static Electricity, in X-ray and Therapeutic Uses. By S. H. Monell, M.D., founder and chief of the Brooklyn Post-Graduate School of Clinical Electro-Therapeutics and Roentgen Photography, etc., etc. Illustrated. Published by William Beverly Harrison, 3 and 5 W. 18th St., N. Y., 1897.

For enthusiasm and optimistic prophecy, we have nevei read so refreshing an effort.

The work is truly interesting and instructive. Though we are not qualified sufficiently to judge of the merits of the static current in Roentgen photography, yet we are prepared to accept the author's statement, knowing the marvelous static current as we do.

The parts treating of the therapeutics of the static current are as orthodox as need be.

Altogether the work commends itself; especially the chapters on X-ray photography. The publishers have just cause to be proud of a work so neat, and of such good material. H. H.

---

HYSTERIA AND CERTAIN ALLIED CONDITIONS. By George J. Preston, M.D., Professor of Diseases of the Nervous System, College of Physicians and Surgeons, Baltimore, etc., etc., etc. Illustrated. Published by P. Blakiston, Son & Co., Philadelphia, 1897.

Though not teeming with originality, or claiming especial distinction for individual merit, this work is valuable. The author has industriously collected much that is ancient and modern.

The historical sketch is most interesting, and the chapter on viscera and vasomotor disturbances are to the reviewer's mind, especially meritorious.

The great good sense of the author in placing much more stress on the value of suggestion, hydrotherapy, massage, isolation and rest, rather than in drugs, meets fully with our commendation.

We think all medical men can read the book with benefit, and find it conservative and *honest.*

The publishers have given good paper and pleasing print.

H. H.

---

ELEMENTARY PRINCIPLES OF ELECTRO-THERAPEUTICS. For the use of Physicians and Students, with 145 Illustrations. Prepared by C. M. Haynes, M.D. Revised Edition. Chicago. The W. F. Keener Co., 1896.

We have received a copy of this book from the McIntosh Battery and Optical Co., Chicago.

It is both scientific and practical. Everything ordinarily needful to know about Electricity and its uses is told. The introduction gives us an account of the earliest observation of this force, and the last pages deal with Radiography, the latest. It is a plainly written and very interesting and valuable book, and one well worth possessing. M. B. H.

